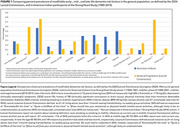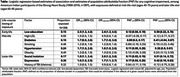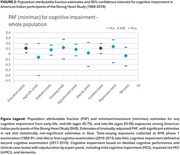# Population attributable fraction for dementia in American Indians

**DOI:** 10.1002/alz70861_107947

**Published:** 2025-12-23

**Authors:** Astrid M Suchy‐Dicey, Kristoffer Rhoads, Thomas J Grabowski, Eric B Larson, Spero Manson, Dedra Buchwald, W T Longstreth

**Affiliations:** ^1^ Huntington Medical Research Institutes, Pasadena, CA USA; ^2^ University Of Washington, Seattle, WA USA; ^3^ University of Washington, Seattle, WA USA; ^4^ Department of Medicine, University of Washington, Seattle, WA USA; ^5^ University of Colorado Anschutz Medical Campus, Aurora, CO USA

## Abstract

**Background:**

The Lancet Commission estimated 45% of dementia cases are collectively attributable to 14 modifiable risk factors (education, hearing loss, head injury, hypertension, heavy alcohol use, obesity, smoking, depression, social isolation, inactivity, diabetes, and air pollution), with assumption that this population attributable fraction (PAF) effect is consistent across populations. However, structural, environmental, cultural, and health contexts may modify factor prevalence, timing of effect, and cross‐factor interactions, especially for heavily‐burdened groups such as American Indians.

**Method:**

The Strong Heart Study recruited middle‐aged American Indians from 13 Tribes in the Southwest, Southern Plains, and Northern Plains in 1989‐91 (*N* =3516). In 2010‐13 (*N* =818) and again in 2017‐19 (*N* =403), an ancillary study recruited surviving participants (85% and 76%, respectively) for detailed neurology examinations. Mild cognitive impairment (MCI) and dementia was adjudicated by consensus panel based on change in cognitive performance over time, according to clinical standards. Logistic regression models supplemented by Gordis method calculated PAF for 10 of the 14 original Lancet factors for cognitive outcomes.

**Result:**

American Indian populations have very different patterns of risk factor prevalence than the populations in the Lancet report, with lower prevalence of dyslipidemia, physical inactivity, heavy alcohol use, social isolation; and higher prevalence of depression, head injury, diabetes, smoking, hypertension, and obesity. Significant PAF findings for MCI and dementia included early‐life education (PAF 12%); mid‐life diabetes (9%), hypertension (11%); late‐life isolation (PAF 11%), depression (17%). Other Lancet Model factors had no risk association.

**Conclusion:**

These findings collectively demonstrate important differences across populations in PAF for dementia. In the Lancet report, 45% of PAF for dementia was attributable to 14 factors; in this study of older American Indians, education, diabetes, hypertension, depression, and isolation alone accounted for 60% of PAF, with other factors not associated. However, more research is needed to understand these unique patterns: especially timing of risk exposure, risk factor burden and interactions, and likelihood to survive to older‐age. Meanwhile, public health programs to address these institutional or structural early‐life exposures, vascular risks at mid‐life, and psychological health in later‐life may provide appropriate targets for reducing risk in American Indian communities.